# The impact of cyberbullying victimization on social anxiety among college students: the chain mediating roles of fear of negative evaluation and rumination

**DOI:** 10.3389/fpsyg.2026.1858777

**Published:** 2026-08-11

**Authors:** Jingying Sha, Zeduo Yang

**Affiliations:** School of Criminology, People’s Public Security University of China, Beijing, China

**Keywords:** college students, cyberbullying victimization, fear of negative evaluation, rumination, social anxiety

## Abstract

**Objective:**

Based on the Clark and Wells cognitive model of social anxiety, this study constructed a chain mediation model to explore the potential mechanisms underlying the association between cyberbullying victimization and social anxiety among college students.

**Methods:**

A total of 3,710 college students were surveyed with the Cyberbullying Victimization Scale, the Brief Fear of Negative Evaluation Scale, the Ruminative Responses Scale, and the Interaction Anxiousness Scale; after data screening, 3,625 valid responses were analyzed.

**Results:**

(1) Significant positive correlations were found among all pairwise combinations of cyberbullying victimization, fear of negative evaluation, rumination, and social anxiety. (2) Cyberbullying victimization significantly and positively predicted social anxiety among college students. (3) In the process by which cyberbullying victimization predicts social anxiety, fear of negative evaluation and rumination not only served as independent mediators but also jointly constituted a chain mediation pathway.

**Conclusion:**

Cyberbullying victimization predicted social anxiety among college students both directly and indirectly through the chain mediating pathway of fear of negative evaluation and rumination.

## 1 Introduction

Social anxiety refers to the significant and persistent fear that individuals experience in social or performance situations, accompanied by concerns about being negatively evaluated by others or exhibiting embarrassing behaviors (American Psychiatric Association, 2013). College students with higher levels of social anxiety are more likely to experience academic difficulties, interpersonal impairments, and occupational maladjustment (Aderka et al., 2012). From the perspective of cognitive-behavioral theory, negative life events and individuals’ cognitive processing styles play a critical role in the development of social anxiety (Clark and Wells, 1995). As the internet has become an important domain for social interaction, the association between cyberbullying experiences and mental health has become increasingly prominent (Kowalski et al., 2014). It is therefore important to examine how cyberbullying victimization and related cognitive factors are associated with social anxiety among college students.

Cyberbullying victimization refers to repeated exposure to deliberate aggressive behaviors perpetrated by others through electronic communication means (e.g., social media, instant messaging), in which victims often find it difficult to effectively defend themselves (Smith et al., 2008). The General Aggression Model posits that cyberbullying victimization, as a negative situational input, can shape individuals’ cognitive and emotional states (Kowalski et al., 2014). Empirical studies of college students have found that cyberbullying victimization is positively associated with social anxiety (Aparisi et al., 2025; Lam et al., 2022). In a latent class analysis of Spanish adolescents, Martínez-Monteagudo et al. (2020) found that the degree of cyberbullying involvement was associated with differences across all dimensions of social anxiety, with the high-cyberbullying group showing elevated social avoidance and distress; a longitudinal meta-analysis in children and adolescents further linked cyberbullying victimization to broader internalizing symptoms, including anxiety and depression (Lee et al., 2025). Therefore, this study proposes Hypothesis 1 (H1): Cyberbullying victimization positively predicts social anxiety among college students.

Although existing research has confirmed the positive predictive association between cyberbullying victimization and social anxiety, several gaps remain. First, prior work has focused primarily on traditional face-to-face bullying (Campbell et al., 2013), whereas research incorporating cyberbullying victimization as an antecedent within cognitive models of social anxiety in the digital age is limited; because cyberbullying has distinct features such as anonymity, wide dissemination, and persistent accessibility (Kowalski et al., 2014), its cognitive and emotional correlates may differ from those of traditional bullying. Second, most studies have examined only a single mediator (e.g., depression, self-esteem) (Wang et al., 2019), rather than the specific transmission pathways from a cognitive-processing perspective. Third, empirical studies have predominantly used Western adolescent samples with relatively small sizes (Lam et al., 2022), and large-scale studies of Chinese college students are scarce; as the collectivist orientation and the concept of “face” in Chinese culture may heighten sensitivity to others’ evaluations (Hofstede, 2001), examining these associations in a Chinese context is of particular value.

The cognitive model of social anxiety proposed by Clark and Wells (1995) holds that fear-based cognitions about negative evaluation occupy a central position in the onset and maintenance of social anxiety. Fear of negative evaluation refers to individuals’ concern about negative evaluation from others and fear of rejection in social situations (Watson and Friend, 1969) and has been identified as a primary factor underlying social anxiety (Rapee and Heimberg, 1997). Gao et al. (2025) reviewed evidence for a stable positive association between fear of negative evaluation and social anxiety. Recent evidence further shows that fear of negative evaluation mediates the link between social exclusion and social avoidance (Zhu et al., 2025). Because cyberbullying victimization is essentially a form of online social exclusion, this study proposes Hypothesis 2 (H2): Cyberbullying victimization indirectly predicts social anxiety through the mediating pathway of fear of negative evaluation.

Rumination, another potential pathway, refers to repetitive, passive thinking about negative events and the emotions they evoke, without active problem solving (Nolen-Hoeksema, 1991). The Clark and Wells (1995) model identifies post-event rumination as a key process maintaining social anxiety. In a systematic review and meta-analysis of 35 studies (10,082 participants; clinical and non-clinical samples), Edgar et al. (2024) reported a moderate, significant positive correlation between post-event rumination and social anxiety (*r* = 0.45, *p* < 0.001). In a prospective study of 565 college students, Feinstein et al. (2014) found that cyberbullying victimization predicted increased rumination. Accordingly, this study proposes Hypothesis 3 (H3): Rumination mediates the association between cyberbullying victimization and social anxiety among college students.

**Figure 1:**
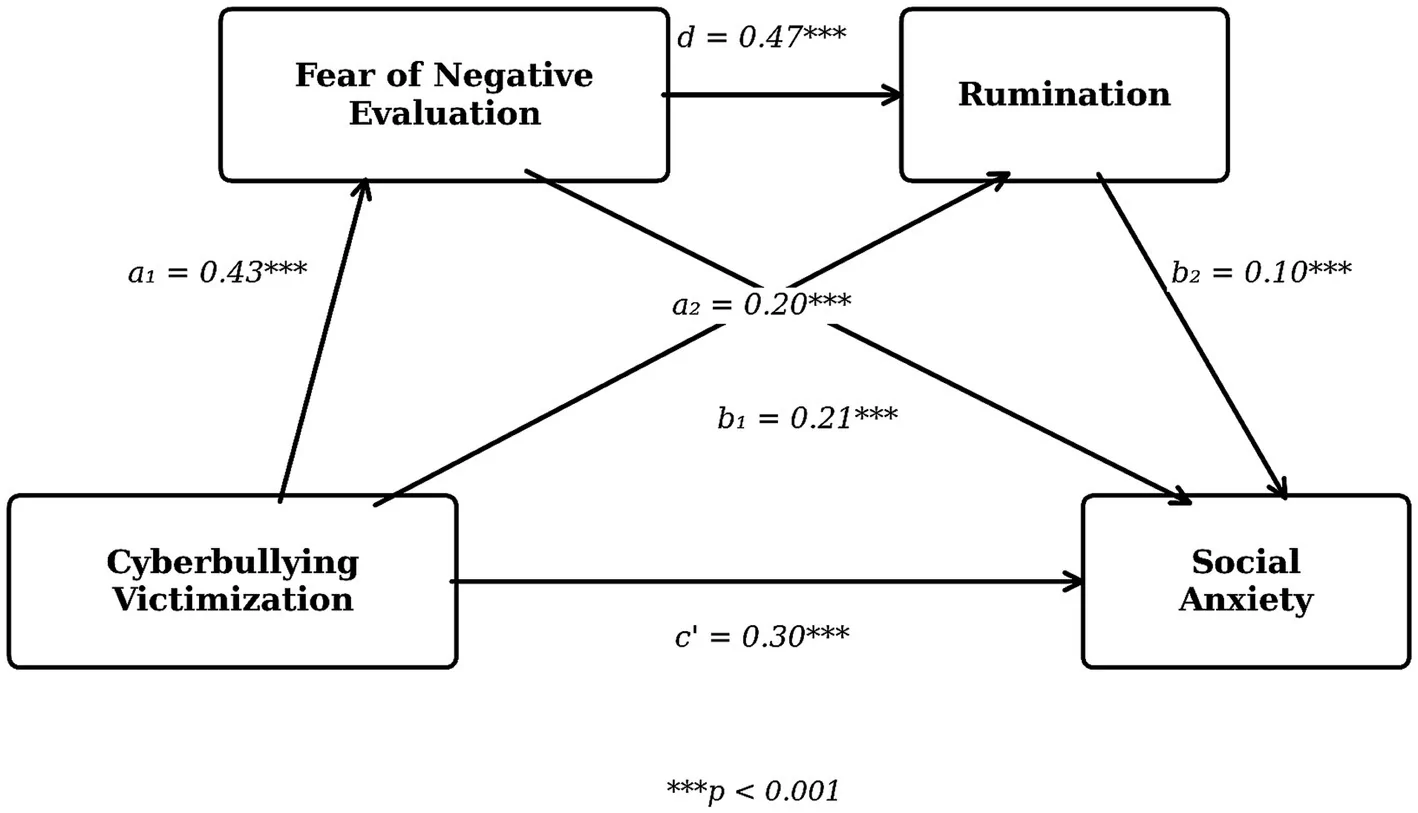
Proposed hypothesis model. H1: Cyberbullying victimization → social anxiety (direct); H2: Via fear of negative evaluation; H3: Via rumination; H4: Via the chain of fear of negative evaluation and rumination. *p* < 0.001.

Fear of negative evaluation and rumination are not independent; they may constitute a chain mediating pathway. The Clark and Wells (1995) model suggests that fear of negative evaluation leads individuals to monitor their performance and engage in excessive post-event rumination (Wong and Moulds, 2009); Modini et al. (2018) validated this pathway in clinical samples, and, in a study of 647 adolescents, Tsarpalis-Fragkoulidis et al. (2022) found that fear of negative evaluation positively predicted rumination. Accordingly, this study proposes Hypothesis 4 (H4): Cyberbullying victimization sequentially predicts social anxiety through the chain mediating pathway of fear of negative evaluation and rumination. In summary, based on the Clark and Wells (1995) model, this study constructed a chain mediation model to examine the association between cyberbullying victimization and social anxiety among college students and the chain mediating roles of fear of negative evaluation and rumination (see Figure 1).

## 2 Materials and methods

### 2.1 Participants

A convenience sample of college students was recruited and surveyed online at universities in the Beijing region. A total of 3,710 students were surveyed; after excluding invalid questionnaires (response time under 3 min and highly patterned / low-variability responding), 3,625 valid questionnaires were retained, yielding an effective response rate of 97.7%. Among the participants, 2,833 were male (78.2%) and 792 were female (21.8%); 1,937 were freshmen (53.4%), 1,064 were sophomores (29.4%), 418 were juniors (11.5%), and 206 were seniors (5.7%). The mean age was 19.15 years (SD = 1.16). The study was reviewed and approved by the Academic Committee of the School of Criminology, People’s Public Security University of China (Ethics Review Approval No. [2026]-003). All participants provided informed consent electronically before beginning the questionnaire.

### 2.2 Measures

Cyberbullying Victimization Scale. The Cyberbullying Victimization Questionnaire developed by Li (2015) was used, which contains 9 items on a single dimension measuring different forms of cyberbullying victimization. Participants indicated the frequency of cyberbullying victimization over the past 3 months on a 5-point Likert scale from 1 (never) to 5 (several times a week), with higher scores indicating more severe victimization. Cronbach’s *α* was 0.85.

Social Anxiety Scale. The Chinese version of the Interaction Anxiousness Scale (IAS; Peng et al., 2004) was used to measure social anxiety. This unidimensional scale contains 15 items rated on a 5-point Likert scale ranging from 1 (not at all characteristic of me) to 5 (extremely characteristic of me), with higher scores indicating higher social anxiety. Cronbach’s *α* was 0.84.

Brief Fear of Negative Evaluation Scale. The Chinese version of the Brief Fear of Negative Evaluation Scale (BFNES; Chen, 2002) was used to measure fear of negative evaluation. The scale comprises 12 items rated on a 5-point Likert scale from 1 (not at all characteristic of me) to 5 (extremely characteristic of me), with items 2, 4, 7, and 10 reverse-scored; higher scores indicate greater fear of negative evaluation. Cronbach’s α was 0.83.

Ruminative Responses Scale. The Chinese version of the Ruminative Responses Scale (RRS; Han and Yang, 2009) was used. The scale contains 22 items comprising three dimensions (symptom-focused rumination, reflective pondering, and brooding), rated on a 5-point Likert scale from 1 (not at all characteristic of me) to 5 (very characteristic of me), with higher scores indicating higher rumination. Cronbach’s α was 0.92.

### 2.3 Data analysis

Descriptive statistics, internal-consistency reliability, and zero-order correlations were computed in SPSS 26.0. The chain mediation model was tested with the PROCESS macro (Model 6) for SPSS 26.0; the significance of the direct and indirect effects was evaluated using bias-corrected bootstrap 95% confidence intervals based on 5,000 resamples, with an effect considered significant when its interval excluded zero. The factorial validity of each measure was examined by confirmatory factor analysis in Mplus 8.3 with maximum likelihood estimation. Additional diagnostics (common method bias, multicollinearity, normality, power, and robustness) are reported in the Supplementary material.

## 3 Results

### 3.1 Common method bias test

Harman’s single-factor test was used to examine common method bias. The results revealed six factors with eigenvalues greater than 1, with the first factor explaining 24.94% of the total variance (less than 40%), indicating no serious common method bias.

### 3.2 Descriptive statistics and correlation analysis

Pearson correlation analysis revealed significant positive correlations among all pairwise combinations of cyberbullying victimization, social anxiety, fear of negative evaluation, and rumination. Descriptive statistics and correlations are presented in Table 1, and item-level descriptive statistics for all items, together with a bar chart of variable means with error bars, are provided in the Supplementary material.

**Table 1:** Descriptive statistics and correlation coefficients.

Variable	*M ± SD*	1	2	3	4
1. CBV	2.50 ± 0.75	1			
2. SA	3.00 ± 0.49	0.67***	1		
3. FNE	2.60 ± 0.63	0.51***	0.60***	1	
4. RUM	2.39 ± 0.73	0.46***	0.52***	0.58***	1

CBV, cyberbullying victimization; SA, social anxiety; FNE, fear of negative evaluation; RUM, rumination. ***p < 0.001.

### 3.3 Mediation effect analysis

**Figure 2:**
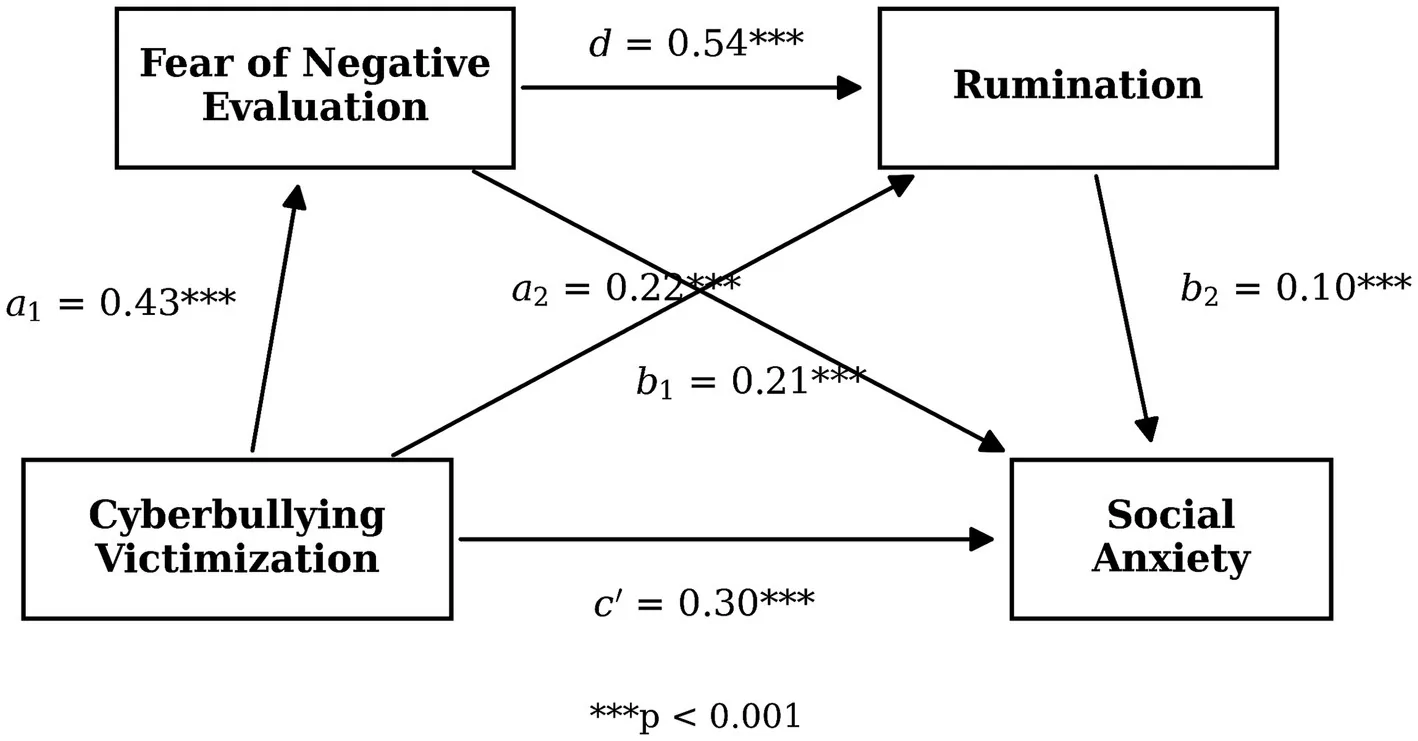
Chain mediation model linking cyberbullying victimization and social anxiety among college students, with fear of negative evaluation and rumination as sequential mediators. *p* < 0.001.

The PROCESS macro (Model 6) for SPSS was used to examine the chain mediation effects of fear of negative evaluation and rumination, with 5,000 bootstrap resamples. All path coefficients were significant (*p* < 0.001). The total effect of cyberbullying victimization on social anxiety was significant (effect = 0.44, *p* < 0.001). With fear of negative evaluation and rumination included, the direct effect remained significant (effect = 0.30, *p* < 0.001). The total indirect effect was 0.14, accounting for 31.05% of the total effect, through three pathways: Pathway 1: Cyberbullying victimization → Fear of negative evaluation → Social anxiety (effect = 0.09, 20.83% of the total effect); Pathway 2: Cyberbullying victimization → Rumination → Social anxiety (effect = 0.02, 5.02%); Pathway 3: Cyberbullying victimization → Fear of negative evaluation → Rumination → Social anxiety (effect = 0.02, 5.20%). Confidence intervals are presented in Table 2 and the path coefficients in Figure 2.

**Table 2:** Chain mediation effect analysis of cyberbullying victimization predicting social anxiety.

**Pathway**	**Effect**	**Proportion**	**95% CI**
Direct effect: CBV → SA	0.30	68.95%	[0.29, 0.32]
Indirect: CBV → FNE → SA	0.09	20.83%	[0.08, 0.10]
Indirect: CBV → RUM → SA	0.02	5.02%	[0.02, 0.03]
Indirect: CBV → FNE → RUM → SA	0.02	5.20%	[0.02, 0.03]
Total indirect effect	0.14	31.05%	[0.13, 0.15]

CBV, cyberbullying victimization; SA, social anxiety; FNE, fear of negative evaluation; RUM, rumination; CI, confidence interval.

## 4 Discussion

Based on the Clark and Wells (1995) cognitive model, this study examined how cyberbullying victimization is associated with social anxiety among 3,625 college students. All four hypotheses were supported: cyberbullying victimization positively predicted social anxiety; fear of negative evaluation and rumination each mediated this association; and the two mediators jointly constituted a chain pathway. These findings support the applicability of the cognitive model in cyberbullying contexts.

First, cyberbullying victimization predicted social anxiety, consistent with prior research. In a study of 486 college students, Lam et al. (2022) reported a significant positive correlation between social anxiety and cyberbullying victimization. In a sample of 1,185 Spanish adolescents (aged 12–18), Martínez-Monteagudo et al. (2024) reported that cybervictims showed higher levels of general anxiety, depression, and stress. Using structural equation modeling with 1,368 Spanish college students, Aparisi et al. (2025) found significant associations among cyberbullying victimization, social anxiety, and maladjustment. This result is consistent with the General Aggression Model (Anderson and Bushman, 2002), in which cyberbullying victimization, as a negative situational input, is thought to activate cognitive schemas and negative emotional states. In the present analysis, even after controlling for fear of negative evaluation and rumination, cyberbullying victimization retained a significant direct association with social anxiety (c′ = 0.30, *p* < 0.001).

Second, fear of negative evaluation was a partial mediator, accounting for the largest share of the indirect effect (67.07% of the total indirect effect), consistent with Clark and Wells (1995), who regard fear of negative evaluation as a primary factor underlying social anxiety (Rapee and Heimberg, 1997). Cyberbullying victimization may foster the core belief that “others will evaluate me negatively,” which may lead individuals to over-attend to evaluative feedback. Gao et al. (2025) reported a stable positive association between fear of negative evaluation and social anxiety; such fear may foster interpretive biases toward social cues (Amir et al., 1998), which are in turn associated with social anxiety.

Third, rumination was also a partial mediator. Although its independent effect was small (16.17% of the total indirect effect), its theoretical significance is notable. Edgar et al. (2024) reported a moderate positive correlation between post-event rumination and social anxiety (r = 0.45) in a meta-analysis of clinical and non-clinical samples. In college students, Feinstein et al. (2014) found that cyberbullying victimization predicted increased rumination. In a sample of Chinese college students, Xu and Li (2024) found that relative deprivation and rumination sequentially mediated the association between upward social comparison and social anxiety.

Fourth, fear of negative evaluation and rumination served as chain mediators (16.76% of the total indirect effect), revealing a sequential cognitive process consistent with Clark and Wells (1995). Cyberbullying victimization may first heighten fear of negative evaluation, which is in turn associated with excessive rumination about social events; in a study of 647 adolescents, Tsarpalis-Fragkoulidis et al. (2022) found a positive correlation between rumination and fear of negative evaluation.

Theoretically, this study extends the Clark and Wells (1995) model by incorporating cyberbullying victimization—a digital-era stressor—into its framework, showing that the model applies beyond traditional face-to-face situations. Practically, the findings suggest several entry points for prevention: reducing cyberbullying, applying cognitive restructuring to modify irrational evaluative beliefs (Beck et al., 1979), and using mindfulness-based interventions to reduce rumination (Kabat-Zinn, 1990); integrative cognitive-behavioral therapy targeting both mediators may be especially effective.

### 4.1 Limitations

This study has several limitations. First, the cross-sectional design permits inferences about associations, not causal relationships; longitudinal or cross-lagged designs are needed to establish temporal precedence. Second, only two mediators were examined; other variables (e.g., self-esteem, emotion regulation, social support) may also be involved. Third, the sample was drawn from universities in a single region and was predominantly male, which may limit generalizability. Fourth, all measures were self-reported, and the rumination scale includes depression-related content; future studies should incorporate additional method sources and control for depressive symptoms.

## 5 Conclusion

This study found that cyberbullying victimization predicted social anxiety among college students, both directly and indirectly through fear of negative evaluation and rumination. The direction of these relationships was specified *a priori* on the basis of the Clark and Wells (1995) cognitive model, in which cyberbullying victimization functions as an antecedent stressor and social anxiety as the outcome; because the design was cross-sectional, these directional predictions should be interpreted as theory-based associations rather than established causal effects. These findings enrich cognitive theories of social anxiety and provide empirical evidence for mental health education among college students.

## Data Availability

The raw data supporting the conclusions of this article will be made available by the authors, without undue reservation.
