# Supplementary material for: The impact of cyberbullying victimization on social anxiety among college students: the chain mediating roles of fear of negative evaluation and rumination
Source: Front Psychol. 2026 Aug 11;17:1858777. doi: 10.3389/fpsyg.2026.1858777 (PMC13503522; doi:10.3389/fpsyg.2026.1858777)
Supplement: Supplementary file 1 [file Supplementary_file_1.pdf]

Supplementary Material

The Impact of Cyberbullying Victimization on Social Anxiety Among College Students: The Chain Mediating Roles of Fear of Negative Evaluation and Rumination

Manuscript ID: 1858777

This document consolidates the supplementary statistical analyses reported during peer review. Unless otherwise noted, all analyses were based on the 3,625 valid cases (N = 3,625). The statistics below reflect the corrected values in the final author proof and the second-round response.

1. Data Screening and Common Method Bias

A total of 3,710 questionnaires were collected. Eighty-five cases (2.29%) were excluded under two predefined criteria: 26 cases (0.70%) had a completion time shorter than 3 minutes, and 59 cases (1.59%) showed invariant responding across all items (zero within-person variance). No case met both criteria. The final sample therefore comprised 3,625 valid questionnaires, corresponding to an effective response rate of 97.7%. The online survey required a response to every item before submission, so the retained dataset contained no missing values.

Multivariate outliers were examined using Mahalanobis distance across the four composite scores as a robustness check rather than as an exclusion criterion. Seven cases (0.19%) exceeded the critical value, $\chi^2(4) = 18.47$, $p < .001$. These cases were retained in the main analyses; excluding them in a sensitivity analysis yielded virtually identical direct and indirect effects.

Harman's single-factor test was used to examine common method bias. Exploratory factor analysis of all 58 items yielded six factors with eigenvalues greater than 1, and the first unrotated factor accounted for 24.94% of the total variance, below the 40% criterion. This result indicated no serious common method bias.

2. Construct-Level Descriptive Statistics, Normality, and Correlations

Table S1

Descriptive statistics, normality indices, and zero-order correlations (N = 3,625)

Variable	M	SD	Skew	Kurt.	1	2	3	4
1. CBV	2.50	0.75	0.14	−0.25	—			
2. SA	3.00	0.49	0.71	0.00	.67***	—		
3. FNE	2.60	0.63	0.41	−0.25	.51***	.60***	—	
4. RUM	2.39	0.73	0.98	0.90	.46***	.52***	.58***	—

Note. CBV = cyberbullying victimization; SA = social anxiety; FNE = fear of negative evaluation; RUM = rumination. ***p < .001.

Figure S1

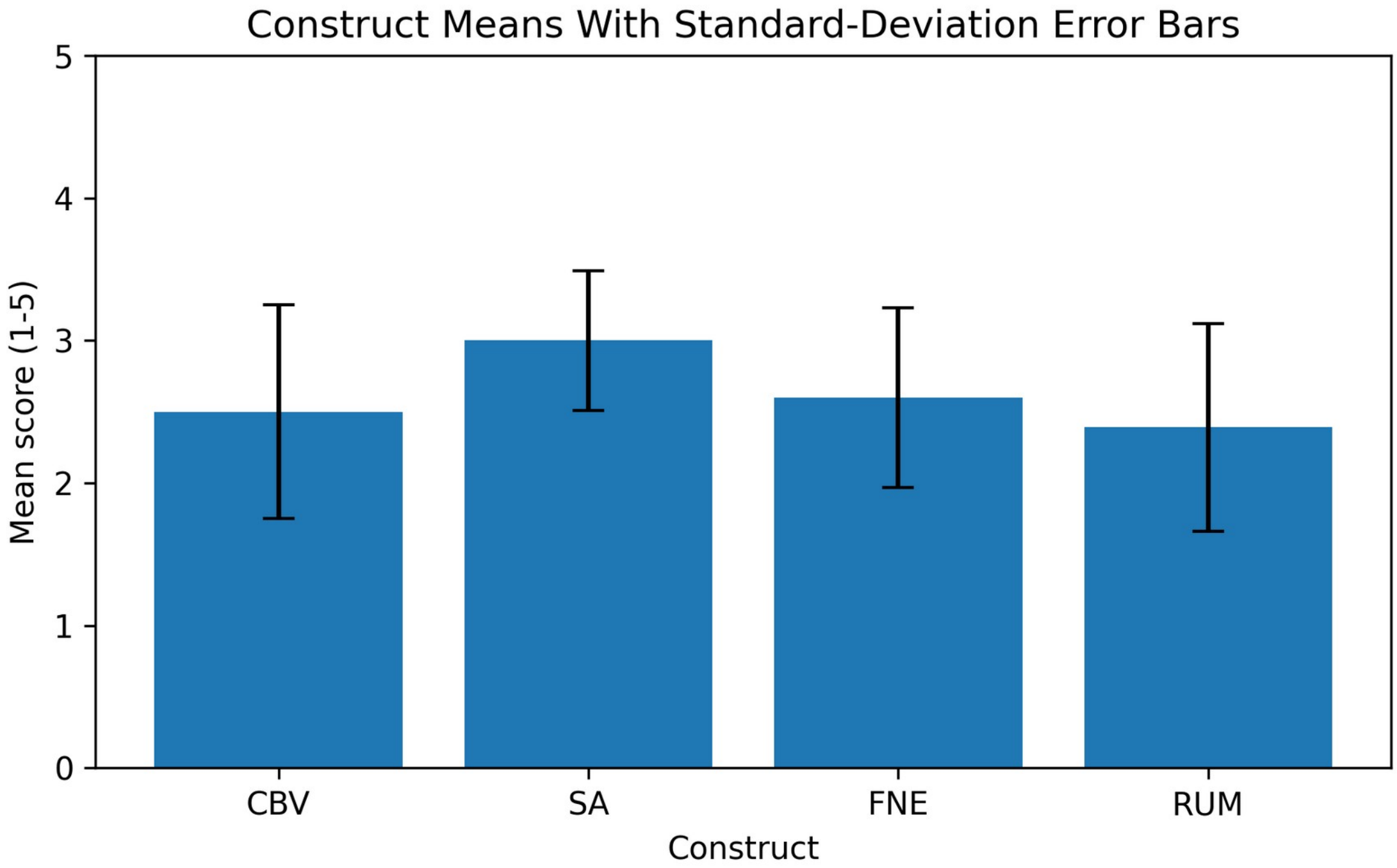

Construct means with standard-deviation error bars.

3. Internal Consistency and Confirmatory Factor Analysis

Table S2

Internal consistency and confirmatory factor analysis fit indices for each scale

Scale	Items	α	CFI	TLI	RMSEA	SRMR	Std. loadings
CBV	9	.85	1.000	1.000	.004	.008	.60–.64
IAS (SA)	15	.84	1.000	1.000	.000	.008	.49–.53
BFNES (FNE)	12	.83	1.000	1.000	.000	.008	.52–.56
RRS (RUM)	22	.92	.922	.913	.056	.041	.56–.62

Note. α = Cronbach's alpha. Fit indices are from single-factor congeneric measurement models estimated by maximum likelihood. All standardized loadings were significant ($p < .001$). Conventional criteria: CFI/TLI ≥ .90, RMSEA ≤ .08, and SRMR ≤ .08.

4. Multicollinearity Diagnostics

Table S3

Collinearity diagnostics for predictors of social anxiety

Predictor	Tolerance	VIF
CBV	.694	1.44
FNE	.590	1.70
RUM	.627	1.59

Note. All VIF values were well below conventional cutoffs of 5 and 10, indicating no multicollinearity concern.

5. A Priori Power Analysis

An a priori power analysis was conducted for the regression-based mediation framework ($\alpha = .05$; three predictors). To detect a small effect ($f^2 = 0.02$) with power = .80, at least 549 participants were required; for a medium effect ($f^2 = 0.15$), 77 participants were required. The observed sample ($N = 3,625$) substantially exceeded these requirements, yielding statistical power approaching 1.00 for the effects examined.

6. Chain Mediation Analysis (PROCESS Model 6)

Table S4

Regression coefficients of the chain mediation model

Path	b	SE	t	p
CBV → FNE (a_1)	0.432	0.012	35.98	< .001
CBV → RUM (a_2)	0.224	0.015	14.89	< .001
FNE → RUM (d)	0.537	0.018	30.05	< .001
CBV → SA, direct (c′)	0.303	0.009	34.54	< .001
FNE → SA (b_1)	0.212	0.011	18.77	< .001
RUM → SA (b_2)	0.100	0.009	10.64	< .001
CBV → SA, total (c)	0.440	0.008	54.50	< .001

Note. Unstandardized coefficients. Variance explained: R^2 = .263 for the FNE equation, .373 for the RUM equation, and .550 for the SA equation.

Table S5

Bootstrap analysis of direct and indirect effects (5,000 resamples)

Effect	Est.	Boot SE	95% CI	Std.	% total
Direct: CBV → SA	0.303	0.009	[0.285, 0.320]	0.462	68.82
Indirect 1: CBV → FNE → SA	0.092	0.006	[0.081, 0.102]	0.140	20.81
Indirect 2: CBV → RUM → SA	0.022	0.003	[0.017, 0.028]	0.034	5.09
Indirect 3: CBV → FNE → RUM → SA	0.023	0.003	[0.018, 0.028]	0.035	5.27
Total indirect effect	0.137	0.006	[0.125, 0.149]	0.209	31.18
Total effect	0.440	0.008	[0.424, 0.455]	0.671	100.00

Note. Bias-corrected percentile confidence intervals were based on 5,000 bootstrap samples; a 95% CI excluding zero indicates statistical significance. Std. = completely standardized effect. All indirect effects were statistically significant.

7. Robustness Check Controlling for Demographic Variables

The direct and indirect pathways were re-estimated while controlling for gender and academic year. The substantive predictors remained significant ($p < .001$), whereas gender and academic year were not statistically significant, indicating that the findings were not driven by the demographic composition of the sample.

Table S6

Social anxiety regressed on predictors and demographic covariates

Predictor	b	SE	p
CBV	0.303	0.009	< .001
FNE	0.212	0.011	< .001
RUM	0.100	0.009	< .001
Gender	−0.005	0.013	.684
Academic year	0.008	0.006	.172

Note. Outcome = social anxiety. Gender was coded 1 = male and 2 = female; academic year was coded 1–4 (freshman–senior).

8. Convergent and Discriminant Validity

Composite reliability (CR) and average variance extracted (AVE) were computed for each scale from the standardized loadings of the single-factor measurement models. All CR values were at least .83, confirming good composite reliability. AVE values were below .50. Because low AVE makes the square-root-of-AVE comparison less informative, discriminant validity was assessed using the heterotrait–monotrait ratio of correlations (HTMT). All HTMT values were below the conservative .85 cutoff, supporting empirical distinctiveness of the four constructs.

Table S7

Composite reliability and average variance extracted for each scale (N = 3,625)

Scale	Items	α	CR	AVE	Std. loadings
CBV	9	.85	.85	.38	.60–.64
IAS (SA)	15	.84	.84	.26	.49–.53
BFNES (FNE)	12	.83	.83	.29	.52–.56
RRS (RUM)	22	.92	.93	.36	.56–.62

Note. CR = composite reliability; AVE = average variance extracted; α = Cronbach's alpha. CR and AVE were computed from standardized loadings. All standardized loadings were significant ($p < .001$).

Table S8

Heterotrait–monotrait ratio of correlations (HTMT)

Construct	1	2	3	4
1. CBV	—			
2. SA	.80	—		
3. FNE	.61	.71	—	
4. RUM	.53	.59	.66	—

Note. CBV = cyberbullying victimization; SA = social anxiety; FNE = fear of negative evaluation; RUM = rumination. All HTMT values were below .85.

Supplementary References

Fornell, C., and Larcker, D. F. (1981). Evaluating structural equation models with unobservable variables and measurement error. Journal of Marketing Research 18, 39–50.

Henseler, J., Ringle, C. M., and Sarstedt, M. (2015). A new criterion for assessing discriminant validity in variance-based structural equation modeling. Journal of the Academy of Marketing Science 43, 115–135. doi: 10.1007/s11747-014-0403-8.
